# A Late Presentation of Right Common Iliac Vein Stent Embolization to the Right Atrium: Important Management Considerations

**DOI:** 10.7759/cureus.59233

**Published:** 2024-04-28

**Authors:** Michael Fragner, Jude Elsaygh, Sudarshan S Srivats, Joon Seok Suh, Kevin Pink

**Affiliations:** 1 Internal Medicine, New York-Presbyterian Brooklyn Methodist Hospital, Brooklyn, USA; 2 Internal Medicine, Catholic Medical Center, Manchester, USA; 3 Internal Medicine, Good Samaritan Hospital, San Jose, USA

**Keywords:** atrioventricular reentry tachycardia, intraoperative cardioversion, tachy-arrhythmia, stent endothelialization, iliac stent migration, stent migration

## Abstract

Stent migration is a rare but serious complication of venous stenting, often presenting with chest pain, shortness of breath, and signs of heart failure. Potential complications include arrhythmia, perforation, and valve destruction. Here we present an asymptomatic patient with a late presentation of right common iliac vein stent migration to the right atrium.

## Introduction

Chronic venous outflow obstruction is a prominent cause of chronic venous disease and has a significant impact on morbidity. It severely impacts quality of life, as it can limit mobility and lead to post-thrombotic syndrome consisting of severe pain, edema, and chronic ulceration with potential infection complications [1,2]. After therapeutic anticoagulation and conservative management, the next step for symptom relief is endovascular intervention (minimally invasive approach utilizing a puncture to gain vascular access) with balloon angioplasty and stent placement to improve vascular patency [2]. 

Seager et al. through a systematic review and meta-analysis of chronic venous disease and deep endovenous stenting were able to show that there was significant improvement in quality of life, severity of cardiovascular disease, and ulcer healing rates due to endovascular intervention. However, there was a reported 0-8.7% major complication rate [3]. Additionally, stent migration was prevalent in the studies analyzed, ranging from 0.9% to 4.3% making this complication rare, but present [3]. Although endovenous stenting is often considered a generally safe procedure, silent migration leading to delayed diagnosis can result in an embedded device not amenable to endovascular retrieval. A multimodality and multidisciplinary approach is required to best manage patients and prevent devastating complications.

## Case presentation

**Figure 1:**
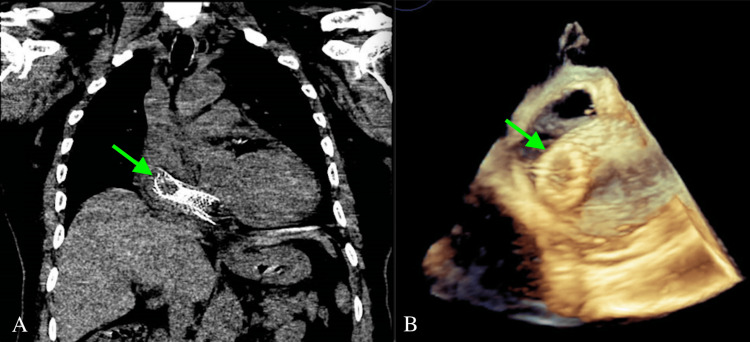
Right Common Iliac Vein Stent Migration to the Right Atrium (A) CT chest and (B) three-dimensional transesophageal echocardiogram (TEE) indicating stent migration.

A 68-year-old male with poor functional status and a history of catatonia with mutism, dementia, and right popliteal deep vein thrombosis and right common iliac vein stenosis with successful stent placement one year prior (16 mm x 100 mm) was transferred from an outside hospital after being found to have embolization of the venous stent to the right atrium. The patient initially presented from his nursing home for tachycardia in the 150s and abdominal discomfort, and he was found to have a urinary tract infection. EMS used adenosine to successfully break the supraventricular tachycardia. The patient denied chest pain on the initial presentation, and a CT abdomen and pelvis was performed (at the outside hospital) revealing a stent present in the RA. He was subsequently transferred for further intervention. CT chest was performed re-demonstrating the migrated venous stent (Figure 1A). A transesophageal echocardiogram (TEE) revealed severe tricuspid regurgitation with a large migrated stent in the RA with the distal end at the inferior vena cava and RA junction. The proximal end of the dislodged stent was at the right atrial appendage with an internal clot. The TEE was done with three-dimensional imaging of the tricuspid valve as shown in Figure 1B. Cardiac surgery evaluated the patient and determined that although the procedure to retrieve the stent would not be complex, the patient’s mental state prevents cardiac surgery due to the patient’s inability to comply with post-operative care.

Endovascular image-guided stent retrieval with interventional radiology was attempted. The first attempt was unsuccessful after being terminated for atrioventricular reentry tachycardia requiring intraoperative cardioversion with cardiac anesthesia. The second attempt was aborted after failed removal due to a high suspicion of stent embedding into the myocardial wall of the RA. This was suspected to be due to late presentation and progressed endothelialization. As the patient was a poor surgical candidate and minimally symptomatic, the decision was made to leave the stent in place and manage it medically. 

## Discussion

Stent migration is influenced by a variety of factors including the stent size and location [4]. It is less commonly seen in upper extremity compared to iliac stents and is more commonly seen in those <60mm in length and diameter <14mm [5]. Sayed et al. encountered 54 cases over a 26-year time period, with only three cases of stent migration of stents with a diameter >14mm, and no cases were reported in stents with a length >100mm [5]. Interestingly, our patient’s documented stent size was 16 mm x 100 mm, making this a rare-sized stent to migrate.

Although the complications of stent migration can be devastating, often patients are fairly asymptomatic with non-specific symptoms. It is reported that up to 41% of such cases are incidental, and these patients present asymptomatically [5]. There are numerous cases that continue to report similar occurrences, implying that the incidence is much higher than is currently being reported [6]. There is a case to be made that our patient was symptomatic with an arrhythmia, given his indication for admission was elevated heart rate. However, he had an additional reason for his tachycardia given his urinary tract infection (likely etiology of tachycardia given resolution after receiving appropriate antibiotics). Nonetheless, it is important to keep a broad differential when considering the etiology of tachyarrhythmia in a patient with known stents.

There are yet to be official guidelines on how to manage venous stent migration, although there is a consensus on imaging surveillance and retrieval endovascularly or surgically [7]. This can often lead to varying approaches and timetables when it comes to intervention. Often stent patency is monitored with ultrasound at six months and one year [7]. Badesha et al., while investigating stent migration incidence and clinical stent patency outcomes, determined that many clinicians monitor migration with different modalities (CT versus duplex sonography) and at a variety of differing intervals [8]. In our patient’s case, it is difficult to ascertain that he was asymptomatic given the history of catatonia and mutism. Given the location of the dislodged stent in a complex location and severe valvulopathy (tricuspid regurgitation), endovascular intervention was warranted. However, the endothelialization of the stent into the myocardial tissue elucidates the theory that the stent had migrated long before presentation.

## Conclusions

Venous stent embolization to the heart is a rare and possibly fatal occurrence. In many instances, however, presentation is delayed due to the asymptomatic nature of the embolization. This points to the necessity for monitoring, which unfortunately in this case did not seem to occur on an outpatient basis. Late presentation portends surgical barriers but can be managed medically with limited symptoms and without complication. Patients with known stents benefit from continued surveillance, and a program including interval echocardiogram, duplex sonography, or CT with defined time intervals may serve to better monitor for migration and improve diagnosis in asymptomatic patients who otherwise go undiagnosed. Leaving stents in place post-migration is not without its potential risks given the possibility of device thrombosis, arrhythmia, and further migration with potential for vascular insult. The decision-making process needs to be carefully undertaken, and we must highlight the importance of a multidisciplinary team approach. Collaboration between multiple specialties including cardiology, cardiac surgery, and interventional radiology is vital in the management of complex cases such as stent migration given the associated high-risk complications.
